# Efficient undergraduate learning of liver transplant: building a framework for teaching subspecialties to medical students

**DOI:** 10.1186/s12909-018-1267-2

**Published:** 2018-07-04

**Authors:** Cheng-Maw Ho, Jann-Yuan Wang, Chi-Chuan Yeh, Yao-Ming Wu, Ming-Chih Ho, Rey-Heng Hu, Po-Huang Lee

**Affiliations:** 1 Department of Surgery, National Taiwan University Hospital, 7 Chung-Shan South Road, Taipei, 100 Taiwan; 2 College of Medicine, National Taiwan University, Taipei, Taiwan; 3 Department of Internal Medicine, National Taiwan University Hospital, Taipei, Taiwan; 4 Department of Medical Education, National Taiwan University Hospital, Taipei, Taiwan; 5 Department of Surgery, E-Da Hospital, I-Shou University, Kaohsiung, Taiwan

**Keywords:** Medical education, Liver transplant, Undergraduate, Efficiency

## Abstract

**Background:**

Liver recipients may develop various diseases after transplant. However, because of inadequate study of liver transplant during undergraduate education, the quality of post-transplant care provided to these patients remains suboptimal. Herein, we introduce an innovative and integrated multimodal pedagogical approach to effectively disseminate key information regarding liver transplant to undergraduate students. The goal is to examine this approach through students’ assessment in multiple dimensions.

**Methods:**

This prospective observational study evaluated student reactions to our pedagogical approach. Fifth-year medical students during the academic year 2015–2016 attended a 2-h session on what nontransplant doctors should know about liver transplants. The pedagogical strategy consisted of an online preclass self-learning exercise, an in-class interactive discussion (facilitated by the class teacher who is a liver transplant specialist to avoid distractions within the short-time frame), and a postclass essay assignment (to integrate and apply concepts). After the class, questionnaires were distributed to individual students to collect data, if returned, concerning the students’ learning experience and feedback to improve teaching quality. Descriptive statistics, Mann-Whitney U tests, chi-squared tests, and McNemar’s tests were used to analyze quantitative data. Qualitative data were content-coded through a descriptive approach using thematic analysis.

**Results:**

Of the 266 attendees, 263 (98.9%) completed the questionnaires and 182 (69.2%) provided comments. Student feedback indicated they “felt better” and “more satisfied” compared with problem-based learning (PBL) (51.0 and 63.1%, respectively) or large-lecture class (92.0 and 88.6%, respectively) approaches. Regarding confidently managing liver transplant patients in future, 80 (30.4%) and 246 (93.5%) students expressed preclass and postclass confidence, respectively (*p* < 0.001). The bell curve of the postclass self-assessment score of learning shifted toward right and became steeper compared with that of the preclass score (*p* < 0.001), suggesting students acquired considerable knowledge. The course was typically perceived to be cost-effective, practical, tension-free, and student-friendly.

**Conclusion:**

This pedagogical approach effectively propagated knowledge concerning liver transplant to medical students, who expressed considerable satisfaction with the approach.

**Electronic supplementary material:**

The online version of this article (10.1186/s12909-018-1267-2) contains supplementary material, which is available to authorized users.

## Background

Medicine is a dynamic and expanding discipline. Studying individual clinical disciplines can be difficult for undergraduate medical students, yielding a low cost-to-performance ratio (i.e., despite intensive learning engagement, facts are easily forgotten because of no hands-on experience and inscrutable general framework) [1]. Many medical students may avoid learning specialized information until they become a specialty resident or often exhibit disorientation, confusion, and lack of overall conceptual framework during clinical rounds with respect to the “tangled” subspecialty disciplines. This leads to a high proportion of newly qualified doctors feeling unprepared to manage patients holistically and facing difficulty with clinical skills and a knowledge deficit [2].

Physicians may be required to consider whether liver transplant is an appropriate option when a patient’s liver function is deteriorating after intervention for other diseases, such as hepatitis B virus (HBV) flare-up in HBV carriers after cancer therapy and severe drug-induced hepatotoxicity during antituberculosis treatment. Physicians typically handle these scenarios without considering the indications or contraindications for liver transplant and the severity of liver dysfunction. Moreover, the number of patients undergoing liver transplant is increasing; as their survival rate improves, these patients may confront common diseases across a broad spectrum of medical fields [3, 4]. Patients have occasionally reported that doctors with a specialty other than liver transplant demonstrate misconceptions or knowledge gaps concerning their illness, resulting in a low quality of care, or in some cases, refusal to provide care [5]. Therefore, there is an unmet need for liver transplant education.

Pedagogical strategies used for teaching medicine include self-directed learning, lecture-based didactic teaching, seminars, informal teaching in clinical settings, problem-based learning (PBL), mentoring, and coaching [6–9]. Most of these approaches require substantial preclass preparation for undergraduates to achieve sufficient efficacy. However, undergraduate class duration remains ever-shortening [10]. Detailed subspecialty-level knowledge is difficult to comprehend without a general overview and hands-on experience, and a majority of medical students generally demonstrate a low retention rate. For subspecialty curriculum development, modern core clinical knowledge must be prioritized, focused on bridging general and basic medicine, and imparted effectively in a short time frame [10].

By using the fundamental concepts of self-paced learning before class, students under a flipped classroom strategy can use the gained knowledge to engage in critical thinking opportunities and application of knowledge through instructor-facilitated learner-centered activities during class [11]. Although a flipped classroom is a promising approach for increasing learners’ motivation and engagement, applying it in a clinical rotation setting may be challenging because of the progressively increasing course content volume and clinical workload and decreasing class duration [11, 12].

In 2014, we identified several key topics and triaged core reading materials on liver transplant as a basis for developing liver transplant curricula [4]. Our pedagogical approach for teaching undergraduates the essential components of liver transplant is a multimodal strategy comprising three components: (1) active preclass self-learning using a study guide, specifically highlighting the essential core knowledge of the discipline as selected by the clinical teacher [10]; (2) an in-class interactive discussion facilitated and guided by the teacher, which avoids distractions during the short time frame; and (3) a postclass essay assignment to integrate and apply concepts. We received positive feedback concerning the pilot use of our approach from medical students. In this paper, we present this innovative and integrated teaching module and report student feedback. We wonder whether this strategy work well for students. The goal is to examine our approach through students’ assessment in multiple dimensions (quantitatively and qualitatively).

## Methods

### Ethics statement

The Institutional Review Board of National Taiwan University Hospital, Taiwan, approved this work and the need for consent to participate was waived.

### Students

This was a prospective observational early-phase study focused on evaluating student reactions to and acceptability of this pedagogical approach. At the National Taiwan University School of Medicine (the top-ranked medical school in Taiwan), fifth-year students (of a 7-year undergraduate medicine course) participate in mandatory surgery rotations during their clerkships. Within the subject of surgery, liver transplant is conducted as a 2-h, small-group (4–6 students/week) course, led by one teacher at the ward meeting room since 2014. Fifth-year students during the academic year 2015–2016 (i.e., September 2015–May 2017) were invited to fill a feedback questionnaire after the class.

### Lesson design and practice

**Fig. 1:**
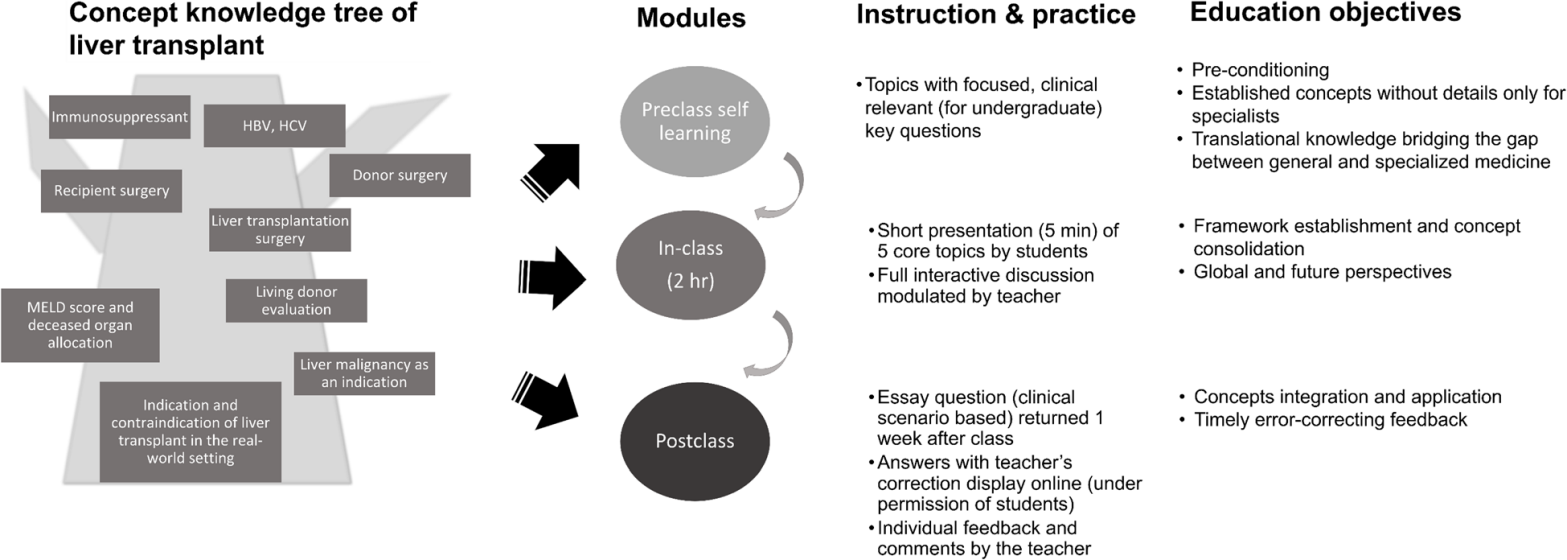
Multimodal pedagogical approach to medical education of liver transplant for undergraduates in a short time frame budget. Abbreviations: HBV, hepatitis B virus; HCV, hepatitis C virus; MELD, Model for End-Stage Liver Disease

The lesson design for liver transplant is illustrated in Fig. 1. Learning materials were screened and narrowed down to form the course content through group discussion among a panel of liver transplant experts and the program director. In total, five “translational” topics concerning liver transplant were selected on the basis of the understanding that the relevant disciplinary knowledge and concepts were well established and sufficient to bridge the gap between general and specialized medicine [4], particularly with respect to what nontransplant doctors are expected to know and what an experienced nontransplant clinician can comprehend through continued medical education. Based on these, students could learn more about the topics and read further regarding the discipline, if interested, after the class. Students were asked to preview the five topics along with the key and focused guiding questions before the class. The students selected one topic each and prepared a 5-min presentation for their small group [13]. The five topics were as follows: (1) Indications and contraindications for liver transplant in the real-world clinical setting; (2) Model for End-Stage Liver Disease scores and organ allocation in liver transplant; (3) Why liver transplant, apart from other solid-organ transplant, is feasible for selected malignancies (e.g., hepatocellular carcinoma); (4) Principle of immunosuppressant use after liver transplant; and (5) HBV and hepatitis C virus (HCV) infection and its management after liver transplant. The teacher prepared suggested references and a concise guide for the students [4]. The learning materials were displayed online since the academic year 2016 [13].

After each presentation, the students were encouraged to discuss the topics in a large-scale conceptual framework, rather than by focusing on details. The teacher would propose thought-provoking questions and concepts during the discussion if the group atmosphere cooled down. The teacher explained students’ questions by illustrating a relevant case scenario to stimulate their thinking, supplement and modify the underlying concepts of topics if inappropriate or missed by the presenters, and guide the discussion in a shortened time frame.

Within 1 week after the class, students were asked to hand in the homework essay, which was a clinical scenario-based open question for concept integration and application—for example, “As a doctor, what do you recommend as the management strategy for a 55-year-old man with hepatitis C viral infection, liver cirrhosis, and hepatocellular carcinoma?” Since the academic year 2016, the students’ answers with teacher’s correction could be displayed online with their permission [14]. This procedure was optional, could be anonymous, and could help other students communicate with the class teacher. Putting corrected work online could also serve as a learning resource for other students. Surgical techniques were not the goal of this course and were not discussed in class. However, observation of a living-donor liver transplant surgery in the operation room was conducted on a different day of the week. Only the liver transplant class adopted this modified pedagogy; most of the other courses employed the PBL method.

### Self-assessment survey and comments: collection of information to improve the quality of and satisfaction with the teaching and learning experience

The questionnaire was formatted on the basis of expert group discussion (liver transplant: CMH, YMW, MCH, RHH, PHL; medical education: CMH, JYW, CCY, PHL) and modified on the basis of the obligatory school curriculum feedback and student feedback surveys from the previous pilot study concerning liver transplant class during the academic year 2014. The self-assessment rating method was based on studies examining learning experience [15–19]. The questionnaire included the following: (1) preclass and postclass self-assessment scores on each of the five topics ranged from 1 (*unfamiliar*) to 10 (*good at it*); (2) most- and least-learned topics; (3) preclass and postclass confidence (prepared mindset) in managing future liver transplant patients; and (4) overall impression of and satisfaction with this course compared with PBL and large-lecture class (Additional file 1: Table S1). A free text response option was included. The questionnaire was returned to the teacher immediately after the class if possible. The teacher answered questions, if any, face to face during the survey.

### Statistical analysis

Data are expressed as the mean (standard deviation [SD]) or number (percentage) where appropriate. Chi-squared test was used to compare independent categorical variables (such as sex, school years, or semesters). McNemar’s test was used for correlated categorical variables (such as future confidence in patient management and preclass vs. postclass). Mann-Whitney U tests or Wilcoxon sign-rank tests were used to compare ordinal variables (such as scores). A two-sided *p* < 0.05 was considered statistically significant. Analyses were performed on SPSS (version 21.0; SPSS Inc., Chicago, IL, USA).

## Results

### Students and response rate

**Fig. 2:**
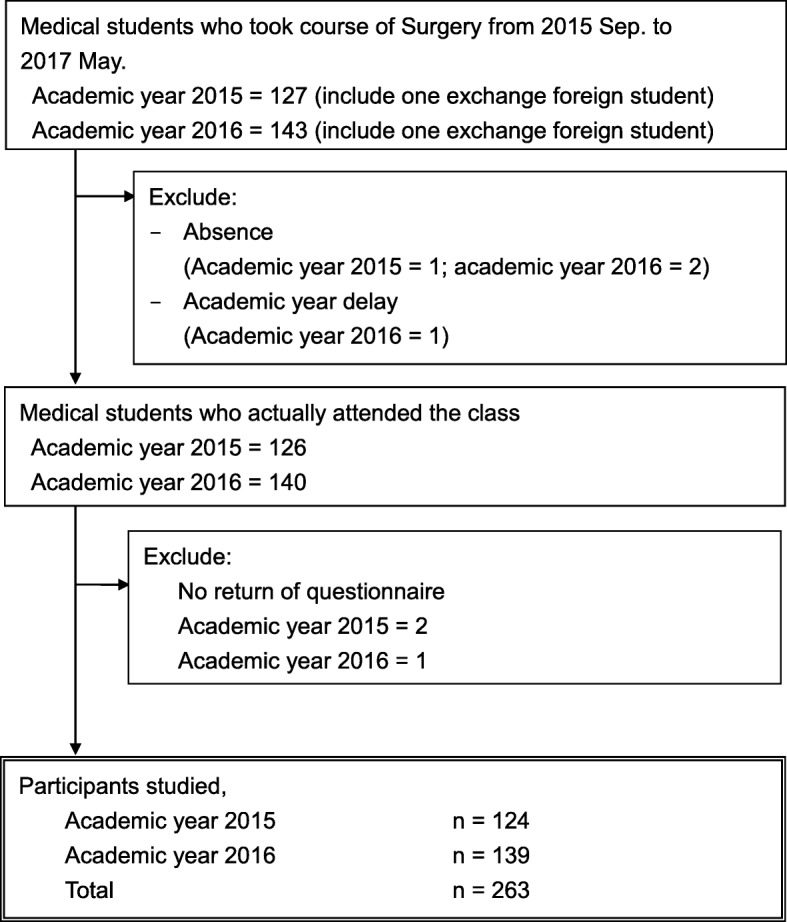
Student participants

In total, 266 medical students attended the class; of them, 263 (98.9%) responded [male: 202 (76.8%)] and completed the questionnaire surveys (Fig. 2).

### Preclass and postclass self-assessment

**Fig. 3:**
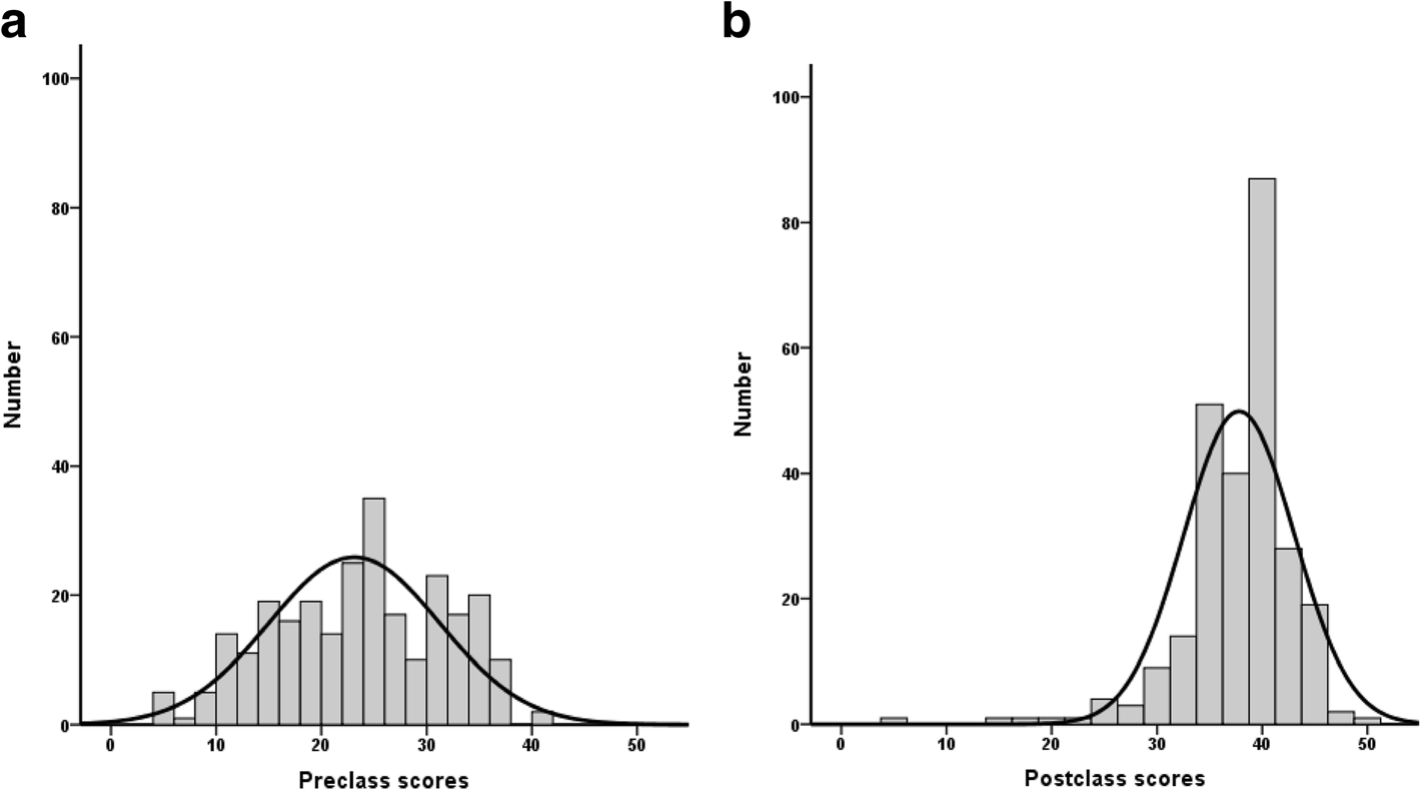
Distribution of preclass (**a**) and postclass (**b**) self-assessed achievement scores

Preclass and postclass self-assessment scores are shown in Fig. 3 and Table 1. The bell curve of the postclass score was right-shifted and steeper compared with that of the preclass score (*p* < 0.001 by Wilcoxon sign-rank test), suggesting that most students acquired considerable knowledge through the class (Fig. 1). Preclass and postclass scores of each topics were also significantly different (*p* < 0.001) (Table 1). Regarding the students’ confidence in their ability to manage liver transplant patients in future, 80 (30.4%) and 246 (93.5%) students expressed preclass and postclass confidence, respectively (*p* < 0.001 by McNemar’s test) (Table 1). Student feedback indicated they “felt better” and “more satisfied” compared with problem-based learning (PBL) (51.0 and 63.1%, respectively) or large-lecture class (92.0 and 88.6%, respectively) approaches (Table 1). The average ΔSelf-assessment score (postclass score − preclass score) was 14.7 (SD: 6.8). Therefore, we divided the participants into two groups according to the ΔSelf-assessment score: high (≥15) and low (< 15). Table 2 lists sex, total scores (preclass and postclass), and future confidence in patient care (preclass and postclass). The two groups were not different in sex, school years, and semesters (chi-squared test). Compared with the low ΔSelf-assessment score group, the high ΔSelf-assessment score group showed significantly lower preclass scores [17.9 (SD: 5.7) vs. 28.2 (SD: 6.7), *p* < 0.001] and lower preclass self-reported future confidence in ability to treat liver transplant patients (22.9% vs. 37.9%, *p* = 0.0015). However, postclass future confidence and total postclass scores did not differ between the two groups (Table 2).

**Table 1:** Characteristics of learning effect in medical students (*n* = 263)

	Preclass	Postclass	*p*
Score (mean, SD)			
Topic 1	4.7 (2.0)	7.6 (1.2)	< 0.001*
Topic 2	4.5 (2.1)	7.7 (1.3)	< 0.001*
Topic 3	5.1 (2.0)	7.8 (1.2)	< 0.001*
Topic 4	4.4 (1.9)	7.4 (1.4)	< 0.001*
Topic 5	4.5 (2.0)	7.5 (1.3)	< 0.001*
Total	23.1 (8.1)	37.8 (5.3)	< 0.001*
ΔScores (mean, SD)			
ΔTopic 1	2.9 (1.6)		
ΔTopic 2	3.2 (1.9)		
ΔTopic 3	2.7 (1.8)		
ΔTopic 4	3.0 (1.6)		
ΔTopic 5	3.0 (1.7)		
ΔTotal	14.7 (6.8)		
Future confidence in patient care			
Preclass (*n*, %)	80 (30.4)		
Postclass (*n*, %)	246 (93.5)		
Comparative evaluation			
Felt better than			
Traditional PBL (*n*, %)	134 (51.0)		
Large-lecture class (*n*, %)	242 (92.0)		
More satisfied than			
Traditional PBL (*n*, %)	166 (63.1)		
Large-lecture class (*n*, %)	233 (88.6)		

ΔSelf-assessment score = postclass score – preclass score

*Wilcoxon sign-rank test

**Table 2:** Medical students stratified by ΔSelf-assessment scores (high [≥15] vs. low [< 15])

	High (*n* = 131)	Low (*n* = 132)	*p*
Sex (male, %)	104 (79.4)	98 (74.2)	0.072*
Academic year (2015/total, %)	66 (50.4)	58 (43.9)	0.324*
Group clustered			0.066*
2015 1st semester (*n*, %)	40 (30.5)	28 (21.2)	
2015 2nd semester (*n*, %)	26 (19.8)	35 (26.5)	
2016 1st semester (*n*, %)	26 (19.8)	39 (29.5)	
2016 2nd semester (*n*, %)	39 (29.8)	30 (22.7)	
Future confidence in patient care			
Preclass (*n*, %)	30 (22.9)	50 (37.9)	0.0015*
Postclass (*n*, %)	120 (91.6)	126 (95.5)	0.558*
Preclass scores, total (mean, SD)	17.9 ± 5.7	28.2 ± 6.7	< 0.001^&^
Postclass scores, total (mean, SD)	38.1 ± 3.9	37.5 ± 6.3	0.822^&^

ΔSelf-assessment score = postclass score – preclass score

*p* value was calculated by either chi-squared test* or Mann-Whitney U test^&^

Table 3 presents topic-specific postclass scores, ΔSelf-assessment scores, and a comparative evaluation. The high ΔSelf-assessment score group had significantly higher ΔSelf-assessment scores in the five individual topics than did the low ΔSelf-assessment score group, but postclass scores did not differ, indicating that all students learned effectively. Compared with a large-lecture class, more than 90% of the participants in both groups had a more favorable impression of this course, and the majority had higher satisfaction. Compared with traditional PBL, roughly half the students in both groups had a more favorable impression of the course, and more than 60% of them reported higher satisfaction (Table 3).

**Table 3:** Topic-specific scores and comparative evaluation of educational effect for medical students stratified by ΔSelf-assessment scores (high [≥15] vs. low [< 15])

	High (*n* = 131)	Low (*n* = 132)	*p*
Postclass scores (mean, SD)			
Topic 1	7.5 (1.0)	7.6 (1.4)	0.052^&^
Topic 2	7.7 (1.1)	7.6 (1.5)	0.775^&^
Topic 3	7.9 (1.0)	7.7 (1.4)	0.953^&^
Topic 4	7.5 (1.2)	7.3 (1.5)	0.673^&^
Topic 5	7.6 (1.0)	7.4 (1.5)	0.634^&^
ΔScores (mean, SD)			
ΔTopic 1	3.9 (1.4)	1.8 (1.1)	< 0.001^&^
ΔTopic 2	4.3 (1.7)	2.0 (1.3)	< 0.001^&^
ΔTopic 3	3.9 (1.6)	1.5 (0.9)	< 0.001^&^
ΔTopic 4	4.0 (1.5)	1.9 (1.1)	< 0.001^&^
ΔTopic 5	4.0 (1.5)	1.9 (1.1)	< 0.001^&^
Comparative evaluation			
Felt better than			
Traditional PBL (*n*, %)	74 (56.5)	60 (45.4)	0.374*
Large-lecture class (*n*, %)	119 (90.8)	123 (93.2)	0.196*
More satisfied than			
Traditional PBL (*n*, %)	82 (62.6)	84 (63.6)	0.694*
Large-lecture class (*n*, %)	113 (86.3)	120 (90.9)	0.337*

ΔScores = postclass score – preclass score

*p* value was calculated by either chi-squared test* or Mann-Whitney U test^&^

Abbreviation: PBL problem-based learning

### Most- and least-learned topics and relation with other factors

**Fig. 4:**
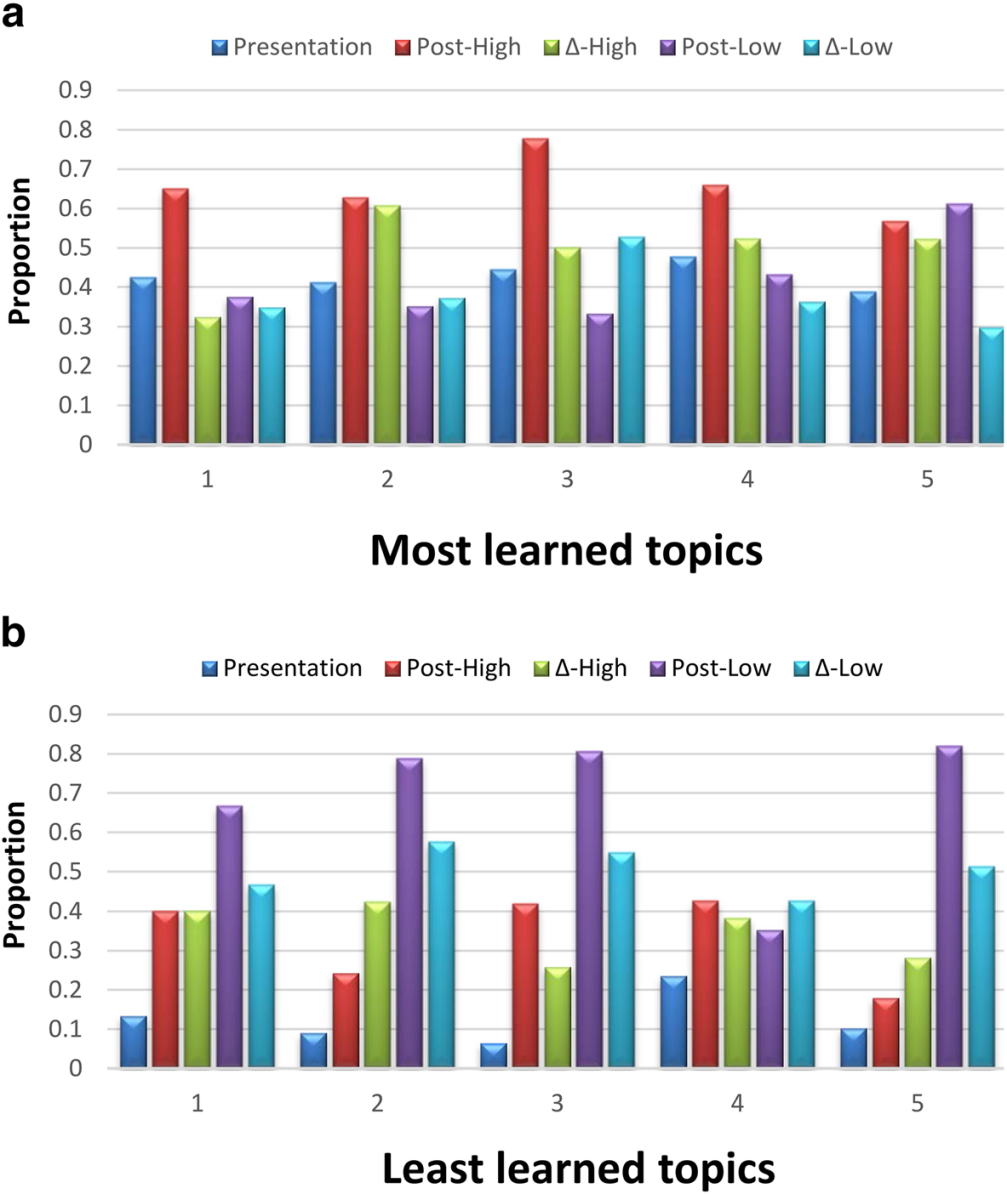
Self-reported most (**a**)- and least (**b**)-learned topics in relation to other parameters. Presentation: The topic is the presentation topic by the student; Post-High: the topic has the highest postclass score by self-assessment; Δ-High: the topic is the one with the greatest improvement between preclass and postclass scores; Post-Low: the topic has the lowest postclass score by self-assessment; Δ-Low: the topic is the one with the least improvement between preclass and postclass scores

In total, 232 (88.2%) and 201 (76.4%) participants reported the most- and least-learned topics, respectively. Topic 5 was the most-learned topic for 65 students, followed by 2 (*n* = 51), 4 (*n* = 43), 1 (*n* = 40), and 3 (*n* = 33), whereas topic 4 was the least-learned topic for 68 students, followed by 5 (*n* = 39), 2 (n = 33), 1 (*n* = 31), and 3 (*n* = 30). Figure 4a and b depicts the most- and least-learned topics, respectively, along with their relation with other factors (presentation topic, highest postclass score, highest ΔSelf-assessment score, lowest postclass score, and lowest ΔSelf-assessment score). In more than 50% of cases, the most-learned topic was the topic with highest postclass scores (Fig. 4a). In more than 60% of cases, the least-learned topics were those with the lowest postclass scores and were usually not the self-presented topic, except for topic 4 (principle of immunosuppressant use in liver transplant) (Fig. 4b). Among students who selected topic 5 (HBV, HCV, and their management in liver transplant) as the most- or least-learned topic, topic 5 was the one with the lowest postclass score in 61.2% (41/67) and 82.1% (32/39), respectively. This suggested that students find topic 5 difficult, despite the self-reported improvement in understanding. This information reflects rapid changes in the literature on HCV management and could also indicate further directions for modification of course content. The average ΔSelf-assessment scores of the topic(s) presented by the students were similar to those of topics not presented by them [2.83 (SD: 1.99) vs. 2.83 (SD: 1.30)].

### Analysis of feedback comments

In total, 182 (69.2%) participants provided feedback comments, classified into seven categories (overall or general, tension and atmosphere in class, learning benefit, study loading and cost-effectiveness, curriculum design, teacher side, and miscellaneous) and are presented in Table 4; source data appear in Additional file 2: Table S2. Among those giving qualitative feedback, 76 (41.8%) responded with comments that could be classified into more than one category. The reactions to the course were mostly positive. Almost all felt their learning experience were better—or at least the same—compared with traditional PBL (Table 4). The course was perceived to be cost-effective, practical, tension-free and student friendly. Students acknowledged the teachers’ dedication with respect to course design and overall practice. Typical examples were identified in the written responses and included the following for learning benefit: “do not need to memorize too many details, but still achieve long-lasting memory without getting confused,” “can connect pieces of knowledge into a whole,” and “thought-provoking experience.” For clinical mindset preparation, students reported the following reactions: “helped me understand what patients want me to know from their perspective,” “emphasized how to solve patients’ problems,” and “correlates with previous experience with postoperative patients.” Some students indicated they would have benefited more from a real case study. A total of 27.5% (50/182) of the students commented specifically on the study workload, curriculum design, and cost-effectiveness. Fifteen students provided comments regarding study workload, and most of them considered the preparation to be “just enough,” “not too much,” “easy,” or “well-balanced between study workload and learning effectiveness.” Compared with preclass self-assessment scores, postclass scores were significantly higher in all categories (Table 4). Overall, students responded that they had highly satisfactory learning experience.

**Table 4:** Feedback comments from students after the course and the overall learning effect

Theme	Category	Example	Impression compared to traditional problem-based learning (better/equal/less)	Total self-achievement scores		
				Pre	Post	p*
Learning experiences	Overall/general (73/182)	Very good; practical; interesting; concise; to the point; impressive; terrific; impressive with discussion and thinking	41/32/0	23.4 ± 8.3	37.9 ± 5.7	< 0.001
	Tension and atmosphere in class (10/182)	Relaxing, low-pressured atmosphere	3/7/0	23.2 ± 7.2	39.9 ± 4.1	< 0.001
	Learning benefit (79/182)	Got a lot; easy to remember; don’t need to know too details; long-lasting memory and not lose track; thought provoking!	47/31/0 (1 not answered)	24.4 ± 7.6	38.6 ± 4.0	< 0.001
	Study loading and cost-effectiveness (15/182)	Well-balanced between loading and learning effectiveness; very high learning effectiveness; had learn something and not too much loading	12/3/0	27.3 ± 9.3	39.1 ± 5.5	< 0.001
Curriculum and teaching	Curriculum design (35/182)	Clear and focused guide and learning objectives for self-study before class; novel webpage; good teaching method, emphasis or utilizable knowledge instead of advanced guidelines; good learning model; very small topic design; systematic discussion	26/9/0	23.9 ± 7.8	39.3 ± 2.8	< 0.001
	Teacher side (39/182)	Nice; patiently; elaborative; enthusiastic	18/20/0 (1 not answered)	23.0 ± 7.6	37.2 ± 6.1	< 0.001
Miscellaneous	Motivation, presentation skill, criticism (20/182)	Hope to learn more about liver transplantation and immunosuppressant prescription; demand high presentation skills; classmates (or I) may prepare/present inappropriately; topic 5 is too heavy to digest	9/11/0	26.1 ± 8.7	37.4 ± 8.5	< 0.001

*Wilcoxon signed-rank test

## Discussion

Our work illustrated a modified multimodal teaching and learning curriculum model facilitating cost-effective liver transplant education for undergraduates. Student feedback indicated a positive impression and high satisfaction for this course model compared with PBL and large-lecture classes. Most students had high postclass confidence in the future care of liver transplant patients. Group performance was consistently assessed by the class and all ward teachers as mostly satisfactory. Qualitatively, most students expressed strong appreciation for this curriculum practice and responded with positive experiences. Overall, our work lays the foundation for larger-scale approaches in other medical schools and application to other disciplines.

The modified method described here is different from other prominent pedagogical approaches. The discipline-based lecture format has a clear preclinical and clinical divide. A vertically integrated curriculum has less overview and framework establishment for each discipline. PBL is time-consuming for students preparing for classes in each expanding subspecialty field that they are unfamiliar with. They often complain about high acute stress for preparation, particularly when many courses using the PBL model were clustered in a short study period. Therefore, Schwartzstein and Rober [20] emphasized the importance of flexible pedagogies.

Confucius, an ancient Chinese teacher, considered that teaching should be individualized and be according to the learners’ aptitude. Although the current trend suggests learning should occur largely outside the classroom [20], students frequently experience lengthy and inefficient preclass preparation, particularly in the ever-specializing field of medicine. Although performance, as evaluated by the class teacher, only demonstrated a borderline significant positive correlation with the self-assessment scores, the modified pedagogical approach presented in this study can work well and has advantages in being overall highly cost-effective, low pressure, and specific, while having increased teacher involvement.

Our model has components of the flipped classroom approach to teaching medical students (preclass preparation activities, particularly when facilitated by concise, readily accessed online tools, and interactive, engaging small-group classroom activities) [21]. This approach seems to enhance lifelong self-directed learning skills [21]. Although there is no “best” universal single pedagogical strategy for every medical student, a curriculum designed with multiple synergistic approaches can help medical students learn in their own style and be more effectively prepared for the future [22].

A paucity of outcome-based empirical studies assessing the effect of social media in medical education exists [23]. However, social media and Internet search engines are rapidly evolving and are having an increasing influence on education. Students are accustomed and adapted to getting quick and short answers to medical knowledge from the Internet, which may require time to be consolidated. Although such a trend seems irresistible, teachers can help. In our model, the curriculum guideline of liver transplant for undergraduates was posted online and relevant learning materials were listed with a clear source [13]. Furthermore, students’ answers to essay questions were posted online for other students’ reference [14]. Given the potential for social media use in medical education [23], more empirical evaluative studies are required to determine educational value.

A limitation of this study is the lack of individualized correlation of students’ self-assessment with future long-term knowledge retention, and professional performance. Students’ self-requirement, expectations, and complacency may have unclear impacts on the self-assessment scores. Objective validation will be addressed in a future study. In addition, the current study does not make a structured comparison between the student experience on a traditional PBL course and our program to determine potential teaching and learning features that can be added to the PBL program. However, most students had positive experience and highly appreciated the curriculum practice. The main goal of our study was to introduce an innovative educational approach, and survey-participating students to gather information, based on which we could further improve the quality of teaching and learning experience rather than simply rendering a pass–fail decision or comparing this strategy and PBL head-to-head (similar to a phase I/II clinical study for a new drug). In light of the positive reactions to this work, future study critically comparing pedagogies is warranted before widely propagating this innovative approach. Nonetheless, we encourage the application of this model in other subspecialties of undergraduate medical education and in teaching newly qualified postgraduate doctors who lack experience.

## Conclusions

In conclusion, we presented a modified multimodal medical teaching experience highlighting medical undergraduate curriculum practice on liver transplant to fulfill the unmet needs of current patient management. The students appreciated the course and benefited greatly. Overall, our efforts provided an example of a pedagogical approach for translational knowledge propagation of liver transplant for future doctors, which can yield high satisfaction and may be applied to other subspecialties.

## Additional files

Additional file 1: **Table S1.** Feedback questionnaire form after class of liver transplantation. (DOCX 17 kb)

Additional file 2: **Table S2.** Descriptive details of qualitative feedback of students’ comments. (DOCX 28 kb)

## Abbreviations

HBV: hepatitis B virus
HCV: hepatitis C virus
PBL: problem-based learning
