# Supplementary material for: Efficient undergraduate learning of liver transplant: building a framework for teaching subspecialties to medical students
Source: BMC Med Educ. 2018 Jul 4;18:161. doi: 10.1186/s12909-018-1267-2 (PMC6032785; doi:10.1186/s12909-018-1267-2)
Supplement: Supplementary file 1 — Table S1. Feedback questionnaire form after class of liver transplantation. (DOCX 17 kb) [file 12909_2018_1267_MOESM1_ESM.docx]

**Table S1. Feedback questionnaire form after class of liver transplantation**

**Self-assessment of learning**

Degrees of comprehension (1-10; 1 *unfamiliar*, 10 *good at it*)

**Preclass Postclass**

1. 1.

2. 2.

3. 3.

4. 4.

5. 5.

In the future, if I meet a liver transplant patient who need my care, I

**Preclass** **Postclass**

□ know □ know

□ am not clear yet □ am not clear yet

how to solve patient’s problems.

I feel that in this class, I learned **most** in Topic_____, and **least** in Topic .

Compared to **traditional problem based learning**,

Impression □ more □ similar □ less

Satisfaction □ higher □ similar □ lower

Compared to **large-lecture class**,

Impression □ more □ similar □ less

Satisfaction □ higher □ similar □ less

Overall, I feel this course…
