# Supplementary material for: Efficient undergraduate learning of liver transplant: building a framework for teaching subspecialties to medical students
Source: BMC Med Educ. 2018 Jul 4;18:161. doi: 10.1186/s12909-018-1267-2 (PMC6032785; doi:10.1186/s12909-018-1267-2)
Supplement: Supplementary file 2 — Table S2. Descriptive details of qualitative feedback of students’ comments. (DOCX 28 kb) [file 12909_2018_1267_MOESM2_ESM.docx]

**Table S2.** Descriptive details of qualitative feedback of students’ comments

| **Single category** | |
| --- | --- |
| **Overall, general (A)** | |
| Great; terrific; very well-organized, very informative, rich source of references; balance with ease and difficulty, easy to understand; to the point, succinct; impressive, adequate pace, highly interactive in class; help us understand when to consider liver transplantation in patients with liver disease; explain the profound things in a simple way, terrific; terrific, understand more of liver transplantation; with key points; very practical; pretty good; good! A helpful course through just simpler PBL-like discussion of liver transplantation; more familiar with the indication, timing and afterward stuffs of liver transplantation; practical and clear; let me understand what should be paying attention to; not bad, know clearer than previously thought; cool; practical and appropriate for M5 level; interesting discussion; clinical correlated knowledge; inspiring, thought provoking; interesting case, realize the clinical problem and how to apply practically; understand who is suitable for transplant and various criteria; focused discussion make everyone understand liver transplantation better; systematic understanding liver transplantation than traditional PBL; impressive about liver transplantation; panoramic view; realize real-world scenario, better than no feedback; realize more solutions to real-world problems; realized details and dynamic changes of criteria; practical information for future care | |
| **Tension and atmosphere in class (B)** | |
| Learned new knowledge and relaxing course without pressure; relaxing and happy learning | |
| **Curriculum design (C)** | |
| Let us know the framework of liver transplantation, do not need to memorize too many details, but still get long-lasting memory and not lose track; capable of learning knowledge through answer search, and solve problems in the future; autonomous study make deep impression, clear topics with clinical implication, helpful into clinic in the future; difficult to present in 5 min, more practical if limit the total number of slides; can shorten the presentation time further, final slide of each topic based on our hospital or case, wants more cases; references have enough depth for M5, case description may be provided before class (but may be misunderstanding if not clearly state the case is for assisting teaching); clear guide of before-class preparation; topic 3 is frequent asked by patients, topic 4 is more like lecture, more different directives may be better | |
| **Learning benefit (D)** | |
| More thorough understanding of liver transplantation, got a lot what was not understood until teacher’s explanation; more understanding of liver transplantation; let me understand what patients want to know from their sides, and learn how to search reference and explain to them in a simple and clear way; got a lot, understand very well of liver transplantation; know more about core questions than usual PBL; learning effect better based on practical clinical experiences; learn a lot through discussion, long-term memory under interactive discussion; got a lot; got a lot, thanks; got quite much; adequate supplementing make one see more when compared to reading guidelines only. Can connect knowledge pieces altogether; generally understand issues about the indication, contraindication, priority and particular liver disease. Can solve problems and thinking through discussion; learn a lot of concepts about liver transplantation, no systemic understanding previously although something already get exposed sometime before, finally have the opportunity to understand systematically; learn a lot; got a lot and learn a lot; more understanding of liver transplantation such as criteria, indication; learn how to make decision, not on small details; deep learning and got a lot from different aspects of liver transplantation by topics study; learn a lot by self-study and much deeper through discussion in class; learn much about transplantation knowledge and also presentation skills, thanks; learn how to explain risk, principle, and benefit of liver transplant to patients; got something through discussion, know more deeply about transplantation; understand every aspect of liver transplant, thank you; got much and practical references; good learn effect; clear guide and references, learn subject and contents; learn a lot relevant knowledge; learn how to manage a transplant candidate and watch for peri-transplant condition; know much about liver transplantation and have sense for future patient care; learn a lot, more understand the evaluation process of liver transplantation; know much about liver transplantation; learn basics and finally know where they are from; learn more if study hard | |
| **Teacher side (F)** | |
| Teacher is nice and explain clearly; teacher is nice; teacher is nice, patient in supplementing details or what we present not enough; thank teacher for mindful preparation; thank teacher for mindful explanation; teacher’s voice is clear and joyful  ; teacher presents clear, interesting and spirit inspiring even we may be in coma state sometimes; thank teacher for supportive explanation even though tired; thanks for supplementing the description; thanks for thorough teacher's preparation and very good at teaching; teacher pay much attention in teaching; information (pre-class reference and in-class supplement) is rich and sufficient; teacher is elaborative | |
| **Miscellaneous (G)** | |
| Point out indication and post-op management, valuable in vedio-assissted, explain inappropriate case indication; demand high presentation skills; understand multiple aspects from criteria such as infection, cirrhosis, malignancy; I may misunderstood the direction of preparation; it is a surprise and not bad to present and discuss in English; may pass on this; want to know more about immunosuppressant prescription in the real-world case; discuss the real-world underlying cause and feel "so why it is”; realize the evolution of treatment | |
| **Multiple categories (E: study loading and cost-effectiveness)** | |
| Interesting and relaxing, learn a lot | A, B, D |
| very good, specific topic organization | A, C |
| Emphasize on how to solve patients’ problems, no need to memorize trivials, good | A, C |
| Good teaching method, emphasis or utilizable knowledge instead of advanced, complex guidelines | A, C |
| Impressive with discussion and thinking | A, C |
| Teacher provides enough references for study in advance for preparation, more clear than before with regard to liver transplantation | A, C |
| Learning from 5 topic guide questions and perspectives of liver transplantation, provide reference in advance, classmates summarize in a short time, teacher supplement, all together make deep impression, real-world case illustration by teacher let us think more and complete learning | A, C |
| Topics are those patients may ask, feel practical, it is good to have suggested references to read in advance | A, C |
| Clear learning objectives, study references provided, better than self-searching without clear mind and guide | A, C |
| Better than teacher one way talking, 1 or 2 cases may be better | A, C |
| Learn a lot, from different perspective, more easy to be digestive; | A, C, D |
| Very informative, learned much what used to be rough. Classmates presents like reporting a paper which distract audience easily, can limit the slides in the future | A, C, D, G |
| Very good, clear questions but workload differs between questions | A, C, E |
| Terrific, references are updated and suitable to selective self-reading, discussion time and content which teacher shared is in good ratio, very impressive | A, C, F |
| Interesting learning stuff, basic but common, very practical | A, D |
| Learn a lot what used to be unclear concept through study references and discussion. Impressive more than big-class lecture | A, D |
| Succinct and to the point, learn efficiently | A, D |
| Succinct and to the point, got more under discussion | A, D |
| Got a lot, simple and effective | A, D |
| Interesting and learn a lot | A, D |
| Full and practical for future clinical practice | A, D |
| Got a lot, feel full and deeply understood liver transplantation | A, D |
| Interesting, know what I did through study reference assigned in advance | A, D, E |
| Informative, learn a lot, easy to prepare | A, D, E |
| Interesting, force myself to read many papers and realize current liver transplantation in Taiwan | A, E |
| Just enough loading and practical topics for real world | A, E |
| Interesting, practical, adequate content depth | A, E |
| Very good, teacher is very patient | A, F |
| Help understanding about liver transplantation stuff; teacher adequate supply real-world application ; practical and informative | A, F |
| Interesting additional remarks by teacher | A, F |
| Very concise, cover many perspective, thought provoking, win-win between teacher and student | A, F, G |
| Ha, great but difficult for me | A, G |
| Cost-effective, quick learning | A, G |
| Great, interesting, hope to learn more about post-liver transplantation care, and transplantation surgery | A, G |
| Discuss many practical issues that patients or even some doctors are not aware of. Thought provoking! | A, G |
| Interesting, reference papers are clear | A,C |
| Novel teaching style, good effect and less pressure than general PBL | B, C, D |
| Relax in class, just enough loading; scared by other teacher who is very fierce, good learning model | B, C, E, F |
| Relaxed and learn a lot | B, D |
| Teacher is knowledgeable and love to share, relaxed atmosphere, got a lot | B, D, F |
| High leaning effectiveness, very appreciated that teacher can guide a class in a relaxed atmosphere | B, D, F |
| Teacher is passionate, realize many transplant-associated problems not only medicine, but also system and environment, low pressure in class | B, D, F |
| Relaxed and enjoyable; much study reports clustered on this week though | B, E |
| Clear study object, got a lot | C, D |
| Systematic discussion, understand every knowledge of liver transplantation from indication to management | C, D |
| Practical learning, better than simple case discussion, the topic about immunosuppressant needs more background knowledge to let discuss smooth | C, D |
| Topics are narrow and not take a lot of time to prepare, but detailed understanding of liver transplantation | C, D |
| Through self-preparation for presentation in class and discussion with classmates, more impressive about relevant topics and learn a lot | C, D |
| Clear learning topics, easy to remember what had learnt, thank you | C, D |
| Learn more under discussion, not enough time to prepare in this week, suggest course modification (looser interval) to achieve better results | C, D, E |
| Very smart topic design. Besides indication/contraindication, the most I learned is the allocation which involves academic issue and ethical issue. In addition, patient psychological and compliance need to be evaluated. Content is very rich and practical. | C, D, E |
| Novel webpage of learning and teaching in class, adequate difficulty | C, E, F, G |
| Real-time solve questions through interaction, deep discussion of 5 learning topics | C, F |
| Suggestive references are terrific and efficient, the topic in discussion is easy to follow, helpful for future learning and thinking. Teacher love to supplement and feedback with students during the discussion which help us learn better. | C, F |
| Teacher supplements much clinical and realistic knowledge, maybe more thoughts appeared when dealing with real patients | C, F |
| Well-balance between loading and learning effect | D, E |
| Tired but got a lot | D, E |
| Had learn something and not too much loading, good | D, E |
| Learn a lot; more impressive when doing self-study although tired; can correlate with experience in previous exposure to a postop patient | D, E |
| Teacher supplements each presenter which makes me feel good, especially some clinical perspectives and viewpoints are not easy to get from internet and book, so I got a lot | D, F |
| Teacher is good at guiding discussion, got a lot | D, F |
| Knowledge is more impressive through discussion, many new updates, teacher supplements a lot, feel technology is advancing which is more acknowledged in PBL than lecture class | D, F |
| Teacher supplement very detailed and learn a lot | D, F |
| Got a lot, although not comprehensive in answering topic questions by classmates, teacher supplements a lot and I learned a lot | D, F |
| Teacher is diligent, clear and lead the discussion, got a lot | D, F |
| Clear teacher’s description, learn much details, thanks | D, F |
| In the first topic, teacher discuss with us about practical issues in the future. Got a lot even future field may not be liver-related disciplines | D, F |
| Learn much, feel that teacher tailorly design the curriculum and read a lot | D, F |
| Funny teacher and realized a lot of the field | D, F |
| Teacher is nice, understand something previous unclear, some contents are explained clearer by teacher than classmates, feel that classmates can talk less | D, F, G |
| Got a lot from reading references, more impressive when handle ourselves, thought provoking, practical clinical questions | D, G |
| Realize the evaluation process and special clinical condition, tips about PowerPoint making and presentation; good learning | D, G |
| Teacher is nice and supplement patiently, topic 5 is a bit too heavy to digest | E, F |
| First topic presented by me is not to the point during my presentation, teacher may describe more clear in advance. Learn a lot and mistakes were corrected. Teacher supplement much knowledge | F, G |
| Teacher is very considerate to list references on the website; more comprehensively when thinking together with other teachers’ class | F, G |

M5, medical year 5; PBL, problem-based learning
